# National recommendations of the Working Group for Postanalytics of the Croatian Society of Medical Biochemistry and Laboratory Medicine: Quality indicators of the postanalytical phase

**DOI:** 10.11613/BM.2026.010503

**Published:** 2026-02-15

**Authors:** Sonja Podolar, Jelena Vlašić Tanasković, Anja Jokić, Lorena Honović, Vladimira Rimac, Jasna Leniček Krleža

**Affiliations:** 1 Medical Biochemistry Laboratory, General Hospital “Dr. Tomislav Bardek”, Koprivnica, Croatia; 2 Department of Laboratory Diagnostics, General Hospital Pula, Pula, Croatia; 3 Department of Medical Biochemical Diagnostics, University Hospital for Infectious Diseases “Dr. Fran Mihaljević”, Zagreb, Croatia; 4 Department of Transfusion Medicine and Transplantation Biology, University Hospital Centre Zagreb, Zagreb, Croatia; 5 Department of Laboratory Diagnostics, Children’s Hospital Zagreb, Zagreb, Croatia

**Keywords:** quality indicators, postanalytical phase, turnaround time, critical results, quality management

## Abstract

Considering the concept of quality as a degree of excellence, the term quality of laboratory work tells us how excellent our results are in all areas of laboratory work. Therefore, quality indicators have been introduced with the aim of monitoring and measuring quality. Quality indicators describe the efficiency of the laboratory process in the form of a numerical value, providing objective evidence of the conformity of the specified laboratory process with respect to predefined criteria. The Working Group for Postanalytics of the Croatian Society of Medical Biochemistry and Laboratory Medicine has decided to describe the necessary steps in designing and monitoring quality indicators, with an emphasis on the quality of the postanalytical and post-postanalytical phase. The main purpose of these recommendations is to facilitate the incorporation of quality indicators into laboratories’ daily routines. Laboratories in the Republic of Croatia are recommended to monitor three quality indicators in the postanalytical phase of laboratory work: turnaround time, withdrawn or retracted laboratory test reports, and notification of critical results. Additionally, two indicators are recommended in the post-postanalytical phase: monitoring issuance of laboratory test reports and monitoring user satisfaction. Harmonising acceptable performance limits and monitoring of the most commonly used quality indicators opens up the possibility of comparisons between laboratories and a uniform quality of laboratory services throughout the healthcare system.

## Graphical abstract

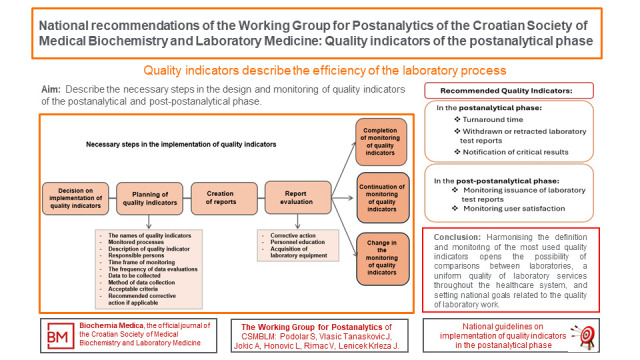

## Highlights

• Quality indicator framework: planning, measuring data, analysing data, implementing improvements

• Recommended quality indicators in the postanalytical phase: turnaround time, errors withdrawn or retracted laboratory test reports, and notification of critical results

• Recommended quality indicators in the post-postanalytical phase: monitoring issuance of laboratory test reports and monitoring user satisfaction

## Introduction

Designing an error prevention mechanism and assessing the quality of laboratory work are priorities for all laboratory professionals. However, ensuring accurate laboratory test results are delivered on time, ultimately leading to correct diagnoses and effective patient treatment, remains a significant challenge. As technological solutions advance in all areas of laboratory work, the demand for effective quality control of laboratory processes has also increased. Quality indicators have been introduced for this purpose. These indicators play an important part in assessing the quality of the total laboratory work (*1*).

Quality indicators describe the efficiency of the laboratory process in the form of a numerical value and provide objective evidence of the conformity of the specified laboratory process with respect to predefined criteria (*2*). Although the result of the quality indicators is an exact value, the procedure of implementing and monitoring the quality indicators can be a challenging task in itself. The lack of time and clear quality objectives often puts laboratory personnel in a situation where they feel responsible for processes that are out of their control, or where monitoring quality indicators does not necessarily lead to quality improvement (*3*). This is particularly evident in the postanalytical phase, such as the reporting of critical results, which extend beyond the laboratory and involve personnel not directly involved in the laboratory work (*4*).

In 2008, the Working Group on Laboratory Errors and Patient Safety (WG-LEPS) of the International Federation of Clinical Chemistry and Laboratory Medicine started a project aimed at creating a list of key quality indicators for the entire laboratory process, enabling laboratories to report their own measured results, modelled on external quality control schemes (*5*). Participation in this program enabled laboratories to measure the quality of their performance in a standardised manner and compare their results with acceptance criteria calculated based on the results reported by all participating laboratories.

Guided by this, the Working Group for Postanalytics of the Croatian Society of Medical Biochemistry and Laboratory Medicine has decided to describe the necessary steps in designing and monitoring quality indicators, with a particular emphasis on the quality of the postanalytical phase. These recommendations aim to make it easier for laboratories to introduce quality indicators into their daily routine. With careful and systematic planning, quality indicators serve as a valuable tool for developing and maintaining the quality of laboratory processes.

## Establishing quality indicators

**Figure 1:**
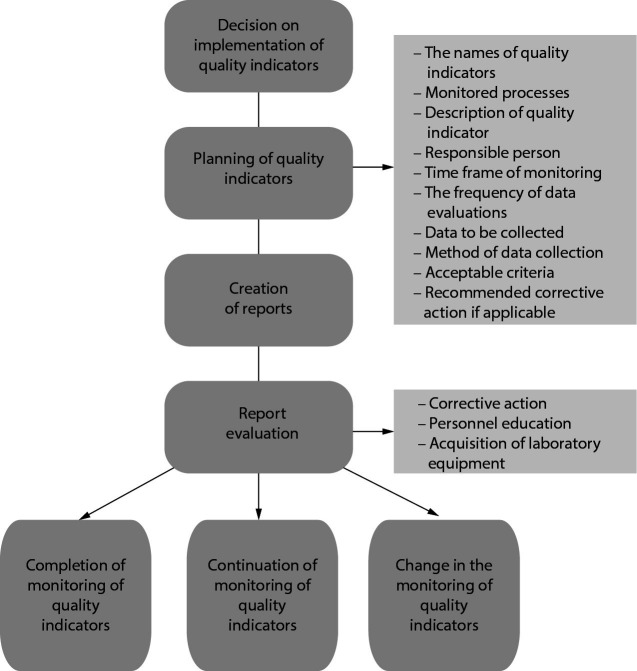
Necessary steps in the implementation of quality indicators.

Quality indicators should be established to assess and monitor the compliance of laboratory processes that require additional attention due to their complexity or processes critical to patient safety. The implementation and monitoring of quality indicators for key laboratory processes are mandatory for every laboratory, in accordance with the standards set by the Croatian Chamber of Medical Biochemists and the ISO 15189 accreditation requirements (*6*, *7*). It is the responsibility of the laboratory manager to decide on the introduction of quality indicators. The further steps of implementation and monitoring, which usually include planning quality indicators, data collection, and analysis, can be delegated to all laboratory personnel (Figure1) (*6*, *7*).

## Planning the implementation of quality indicators

To effectively document all steps involved in planning the implementation of quality indicators, it is recommended to create a planning form. This form should include the following essential elements: a) a name of the quality indicator, b) a laboratory process being monitored, c) a description of the quality indicator, d) individuals responsible for monitoring, e) the data to be collected and the methods for data collection, f) the time interval during which the quality indicator will be monitored, g) the frequency of data evaluations, h) acceptance criteria for the quality indicator with the source mentioned and, i) the propositions of the actions to be taken if the acceptance criteria are not met (Table 1) (*8*, *9*).

**Table 1:** Example of planning of quality indicator: turnaround time for STAT potassium

**Name of quality indicator**	**Turnaround time for stat potassium**
Monitored processes	Preparation of the STAT sample for analysis, transportation of the sample to the analyzer, analysis, review of the test results, additional procedures such as retesting, and release of the laboratory test report
Description of quality indicator	Time elapsed from confirmation of the request from the laboratory to the release of the report for stat potassium
Responsible personnel	Data record: all personnel with LIS authorisation Data assessment: medical biochemist (name)
Time frame of monitoring	Continuously
Data to be recorded/method of recording	1. Time of confirmation of request/LIS 2. Time of report release/LIS 3. Time frame from 1. to 2. in minutes/LIS report
Frequency of reports	Reports: monthly
Acceptable criteria	Time frame: 60 minutes (*13*) Number of reports released after 60 minutes: < 18% (*11*)
Recommended corrective action if applicable	1. Separate place of sample acceptance 2. Mark the sample for visibility during sample preparation 3. Use of the STAT position on the analyzer
LIS - laboratory information system. STAT - position for urgent samples.	

For quality indicators that are continuously monitored in the laboratories, such as turnaround time (TAT), the frequency of data analysis and reporting must be defined. For indicators implemented to obtain specific information about the process (*e.g.*, TAT for manual/semi-automated test analysis to confirm the need for automation of the analysis), a time frame must be established in which specific data is collected, analysed, and reported (*7*, *8*).

It is essential to establish the criteria that quality indicators should meet (*10*). These criteria can be determined based on literature reviews, recommendations from relevant professional societies, standards from interlaboratory comparisons, requirements from laboratory users, or suggestions from equipment and reagent manufacturers.

As previously mentioned, the WG-LEPS criteria define current standards for acceptable laboratory performance limits. Similar to criteria based on biological variability, the 25th percentile indicates high-performance quality, while the 75th percentile reflects a lower level of quality (*11*). Due to the lack of national programs, all Croatian laboratories are invited to use the published criteria as a source of acceptance criteria and participate in this project as the next step in the improvement phase (Table 2). Designing quality indicators according to external quality control schemes or interlaboratory comparisons is the most objective evidence of the quality of laboratory work. However, this approach also has drawbacks. These include the frequent need for manual data collection due to insufficient information systems, the additional workload caused by data entry and report analysis, and, in some cases, the absence of suitable schemes or the use of inadequate criteria (*12*).

**Table 2:** Overview of recommended quality indicators for key processes of the postanalytical phase of laboratory work by Working Group on “Laboratory Errors and Patient Safety”

	**Percentage of laboratory reports released outside the specified time frame**
TAT	TAT for STAT potassium results
	TAT for STAT PT-INR results
	TAT for STAT total blood count results
	TAT for STAT troponin (TnI or TnT) results
	Percentage of STAT potassium results (released after 1h)
Laboratory report	Percentage of revoked laboratory reports
Notification of critical results	Percentage of delayed inpatient critical results
	Percentage of delayed outpatient critical results
TAT - turnaround time. STAT - position for urgent samples. PT-INR - prothrombin time - international normalized ratio. TnI - troponin I. TnT - troponin T.	

## Monitoring of quality indicators

The approach to data collection must be adapted to the laboratory’s capabilities, especially to the available functionalities of the information systems or equipment, as well as to the time commitment of the laboratory personnel involved in the data collection. The person in charge of planning the quality indicators is responsible for creating forms for data collection, training all personnel involved in data collection, and carrying out all other procedures, such as modifying the laboratory information system (LIS) (*9*). Data can be collected manually, where data is collected on a form designed for monitoring quality indicators, or automatically, where data is collected using information systems: LIS, other information systems (*e.g.*, hospital or corporate information systems), or middleware that connects LIS to analyzers.

Using information systems for data collection allows the generation of various reports, and filters can be applied to test results or patient data. Automated data filtering saves time and lessens personnel burden, and the results are often less prone to errors. Data for quality indicators can be collected retrospectively (by filtering data recorded independently of quality indicators monitoring) or prospectively (data collection starts with the introduction of quality indicators) (*8*).

## Creation and evaluation of reports

The report should clearly indicate whether the measured data meet the preset criteria. For easier interpretation, it is recommended to present the results in a table or graph. The interpretation should include a possible explanation for any deviation from the criteria, as well as trend monitoring or comparison with previous results for continuous quality indicators (*8*).

The evaluation of the obtained results is conducted by the laboratory manager and should be included in the laboratory management review (*7*). The laboratory manager decides whether to continue, change, or terminate quality monitoring. Based on the report on the quality indicators, necessary corrective actions can be requested, personnel training can be introduced, or the replacement of laboratory equipment can be planned (*7*).

## Quality indicators of the postanalytical phase

Laboratories are recommended to monitor three quality indicators in the postanalytical phase of laboratory work: TAT, withdrawn or retracted laboratory test reports, and notification of critical results (*13*).

### Turnaround time

Turnaround time is the most frequently monitored quality indicator in laboratories as it most comprehensively describes the laboratory process (*14*). It is also a key quality indicator that physicians and patients, as users of laboratory services, frequently rely on to evaluate the quality of those services (*15*). For most Croatian laboratories, TAT includes the time from confirmation of the laboratory test request in the LIS to the release of the laboratory test report (*16*). Assuming that confirmation of the test request began after the laboratory received the sample, the processes included in this time are preparation of the sample for analysis, transportation of the sample to the analyzer, analysis, interpretation of the test results, additional procedures such as sample dilution or retesting, and release of the laboratory test report. If the confirmation of the test request is followed by sampling in the laboratory, this interval also includes the process of preparing the patient for sampling and the sampling itself.

When monitoring the TAT, a model with two approaches is possible. Laboratories may choose to record the time interval between confirmation of the test request for laboratory tests in the LIS and the release of the laboratory test report. The second approach involves monitoring the number of reports not released within the specified time interval, and the quality indicator is expressed as a percentage of the total number of laboratory reports released (*5*, *17*).

When defining the goals and acceptance criteria for TAT, it is important to evaluate data for emergency and routine samples separately (*18*). For emergency samples, TAT could be monitored for sample or specific tests, as proposed by the WG-LEPS (*5*). The chosen test should best describe all involved laboratory processes. The recommended TAT for emergency testing is up to one hour from the confirmation of the test request to the release of the laboratory test report or test result of the chosen test (*13*).

The turnaround time for routine tests depends on the laboratory’s capabilities, including equipment and personnel, the complexity of the analysis, the test’s cost, and user expectations. Although the TAT of routine tests is rarely the focus of quality assessment of laboratory work, its monitoring better describes the processes that are often skipped for urgent samples, such as non-emergency transportation of samples, either to or within the laboratory, or the storage of samples for tests that are not performed immediately.

The data for the TAT should be recorded daily, including the time of request confirmation and the time of release of the laboratory test report. The recording itself is done automatically if the information systems are used in the laboratory. It is also desirable to customise or upgrade the information systems used to provide the ability to generate reports on the TAT quality indicators. The frequency of recorded data analysis and reports should be adapted to the size and needs of the laboratory. It is recommended that TAT results be analyzed monthly (*6*, *7*). Any deviation from the set criteria should be analyzed, and the cause of the delay in laboratory test reports should be identified. Exceeding the TAT should not always be considered an indicator of compromised quality, but rather the opposite. For example, this may be due to additional tests that must be performed to clarify the results of the first-line tests. However, when evaluating the results and making decisions about further steps, the main goal should be timely patient care (*17*).

### Withdrawn or retracted laboratory test reports

A laboratory test report with incorrect test results can have profound consequences for the patient (*18*). The report with the incorrect result must be withdrawn immediately, and the reason for the error should be identified. If possible, a new sample should be analyzed and the new report with the correct test results should be released. It is necessary to inform the responsible physician about the withdrawal of the report. Both the revoked report and the corrected report must be clearly labelled. Each such adverse event must be recorded and evaluated in the form of a quality indicator (*13*).

The source of error for an incorrectly released laboratory test report can lie in any phase of the laboratory work, including the pre-preanalytical and post-postanalytical phases that take place outside the laboratory (*19*, *20*). Automation of the analytical phase, autovalidation, and the introduction of information systems are just some of the solutions that have helped prevent errors in the laboratory. Regardless, dealing with incorrectly released reports requires the attention of laboratory experts. The minimum data collected for analyzing quality indicators is the total number of incorrectly released test results or the percentage of incorrectly released reports relative to the total number of all released reports (*6*). The records may also include the source of error, which can provide additional information, such as the percentage of each source of error (*21*). The recording of data for this quality indicator should be continuous, with data analysis and reporting of results at least once a year (*6*, *7*).

### Notification of critical results

A critical result is any result that indicates a patient’s health condition is life-threatening or requires immediate medical intervention. Laboratories must have established procedures to report such laboratory test results to the physician as soon as possible (*22*).

A precondition for an efficient reporting of critical results includes a list of tests with associated critical values. Such a list can be based on literature data, as well as on agreed medical decision limits adapted to the patient population or the requirements of the physicians using the laboratory (*23*, *24*). The effectiveness of the process itself depends on the method of reporting. In most cases, critical results are reported by telephone to a healthcare professional involved in the patient’s care, preferably a physician (*13*). Considering the security of medical data and confirmation of receipt, other communication channels can also be used, such as printing laboratory test reports on a designated printer and sending e-mails or messages (*25*).

It is recommended to set an acceptable time frame of 30 minutes for reporting critical results, with the start time implying the time of release of the laboratory test report (*13*). Critical limits can be implemented in information systems to ensure that such results are clearly and timely visible to laboratory personnel. Each laboratory must keep records of reported critical results (*6*, *7*). Records of reported critical results should include the first and last name of the patient whose result is reported, the test with a critical result, the name of the healthcare professional to whom the result was reported, the date and time of the report, and the name of the person who reported the critical result. Records of special notes, such as the impossibility of reporting due to the unavailability of the responsible physician, should also be kept (*7*).

The effectiveness of the notification of the critical results can be assessed by introducing a quality indicator. The data that best describe this quality indicator include: a) the time required to report the critical result, taking the time of release of the laboratory test report as the starting point, or b) the percentage of critical results reported outside the specified time, with the time of release of the laboratory test report as the starting point, or c) percentage of successfully reported results (*14*, *26*).

It is recommended to analyze generated data on critical results and produce a report at least once a year (*7*).

## Quality indicators in the post-postanalytical phase

In addition to those already mentioned, the quality indicators can also refer to other important parameters of the post-postanalytical phase that ensure the principle of comprehensive laboratory management and monitoring the quality of laboratory work.

### Monitoring issuance of laboratory test reports

Although the delivery of laboratory test reports to the client in most laboratories in the Republic of Croatia is ensured by the Central Health Information System, due to problems with system availability and the patient’s personal need to receive laboratory test reports directly from the laboratory, the laboratory reports can be personally collected by patients or sent to the patient’s e-mail. For the latter, a valid consent form must be designed and made easily accessible for patients.

Monitoring the frequency of issuance of laboratory test reports personally collected in the laboratory or the percentage of securely received e-mail reports, including laboratory test reports from referral and collaborative laboratories, as quality indicators, adds value to the quality of the laboratory’s communication with its users. The data obtained can be helpful to the laboratory manager in organizing the work of laboratory personnel, as well as identifying the need to provide additional information to laboratory users. The time intervals for reporting on the proposed quality indicators should be adapted to the size and needs of the laboratory and could be monthly, semi-annually, or annually.

### Monitoring user satisfaction

The basic method of monitoring user satisfaction involves recording compliments, complaints, and suggestions from all users of the laboratory, including patients, clinic personnel, and laboratory personnel (*6*, *7*). The forms for suggestions, compliments, and complaints must be created, made visible, and easily accessible to all users of the laboratory. This quality indicator reflects the mutual communication between the provider and the user and is one of the most important quality indicators. It is therefore recommended that user satisfaction be continuously recorded with a regular monthly report on the above points. The report should also include a record of corrective actions required.

Additionally, laboratory management is required to conduct a user satisfaction survey at least once a year and report the results back to the laboratory staff (*7*). The survey should include both patients and physicians, and should assess user satisfaction with all critical laboratory processes (*27*). The aforementioned processes may include phlebotomy services, TAT, issuance of laboratory test reports, or advisory activities (Table 3). In this way, it significantly contributes to ensuring recognition of and trust in the laboratory’s work results.

**Table 3:** Proposed quality indicators in the post-postanalytical phase

Monitoring issuance of laboratory test reports	Percentage of securely received laboratory test reports by e-mail
	Percentage of issuance of laboratory test reports personally collected in the laboratory
Monitoring user satisfaction	Number of user compliments, complaints, and suggestions
	User satisfaction survey

## Conclusion

Medical biochemical laboratories in the Republic of Croatia are advised to use these recommendations, which provide minimum quality requirements to facilitate the use of quality indicators for certain crucial postanalytical processes. Harmonising the definition and monitoring of the most used quality indicators opens the possibility of comparisons between laboratories, a uniform quality of laboratory services throughout the healthcare system, and setting national goals related to the quality of laboratory work.

To minimise the burden of managing extensive documentation, laboratories should require upgrades from their information system providers that allow for the automatic recording of data monitored in the quality indicator in a standardised manner, while enabling the creation of reports and the ability to evaluate this data through reporting. Ideally, the acceptance criteria for individual quality indicators should be adapted to the needs and requirements of laboratory users. However, it should represent added value and complement the benchmark set by national and international medical group recommendations.

## Data Availability

No data was generated during this study, so data sharing statement is not applicable to this article.
